# Control of Tuberculosis in Massachusetts1Read before the Section on Public Health of the New York Academy of Medicine, November 8, 1895.

**Published:** 1895-12

**Authors:** Frederick H. Osgood

**Affiliations:** Chairman of the Massachusetts Board of Cattle Commissioners


### CONTROL OF TUBERCULOSIS IN MASSACHUSETTS.[1]

BY FREDERICK H. OSGOOD, M.R.C.V.S.,
CHAIRMAN OF THE MASSACHUSETTS BOARD OF CATTLE COMMISSIONERS.

THE time has long passed for discussion as to the cause of tuberculosis, either in man or the lower animals; its prevalence is likewise pretty thoroughly demonstrated. The value of tuberculin as a diagnostic agent in the detection of bovine tuberculosis is clearly recognized; and that this agent has no deleterious effect upon animals free from tuberculosis has also been proven to the satisfaction of all scientific authorities and others who have had practical experience with it. The Cattle Commission of Massachusetts have tested with tuberculin in the past twelve

[1] Read before the Section on Public Health of the New York Academy of Medicine, November 8, 1895.

months about 24,000 animals, and of this number they have destroyed as tuberculous 3450. The percentage of those affected has varied widely in the different sections of the State, 0.9 per cent. in Nantucket to 52 per cent. of those quarantined by local inspectors, and in several large herds as high as 95 per cent. have been found diseased.

The question now arises, What have we accomplished? The experience of the Commission has demonstrated to its satisfaction that where all the diseased animals have been removed at the first examination made with tuberculin, and the premises thoroughly disinfected, the herd has remained healthy, as indicated by subsequent tuberculin-tests made at varying periods of from six to nine months. The provision is made by our Commission, and strictly insisted on, after a herd has been tested, and all diseased animals removed, that the premises shall be thoroughly disinfected, and that no animal shall be introduced into the herd thus cleaned up with tuberculin until such animal has been subjected to the tuberculin-test and proved free from the disease.

A herd familiar to many in this audience is that of the famous Deerfoot Farm, largely composed of thoroughbred Jerseys, the foundation stock having been imported from the Channel Islands. Some two years since the entire herd, consisting of some seventy animals, were subjected to the test; some thirty responded and were destroyed, the autopsies revealing the disease in all stages. The premises were then thoroughly disinfected with live steam, bichloride of mercury, and whitewash. During the past month the entire herd, eighty-six in number, were retested without a single reaction or trace of disease. The veterinary supervision of the herd had been in my hands for some years prior to the test with tuberculin. At previous examinations, conducted about once in six months (never over a year elapsing), I had removed from time to time advanced cases of tuberculosis on physical examination.

I could cite numerous cases similar to the above described. In one of these herds the cow-man in charge is evidently a phthisical subject, yet notwithstanding this fact there has been no case of infection in the interim. In view of these facts, we have felt that in freeing our herds from tuberculosis we are accomplishing some permanent good, for, if the premises are thoroughly disinfected, reinfection is not liable to occur except by the introduction into the herd of an affected animal. It has been urged that the disease is very apt to reappear from the infection through tuberculous attendants, but this is not true either by the history in given districts or by direct experiment. In 1894, our systematic examination conducted on the island of Nantucket (a county famous in the health statistics of Massachusetts for the very large amount of tuberculosis prevalent in the human family) demonstrated the almost entire absence of bovine tuberculosis. In every instance where the cattle were found affected the disease could be traced directly to the importation of tuberculous animals from the mainland.

Laboratory experiments indicate that the bacillus from the human subject is much less virulent than that from the bovine. Comparative microscopical examinations show clearly the rugged, hardy appearance of the bovine, as compared with the slender, bent bacillus of man; and the growth of the cultures together in the same media has demonstrated that the bacillus of the bovine kills out the bacillus from the human subject. In 1864, Villimen, and 1869, Klebs, produced tuberculosis in calves by injecting tuberculosis from man into the peritoneal cavity; but these experiments are directly offset by those conducted by Prof. Theobald Smith, who recently informed me that with the pure culture of the bacilli, procured from the animal pet of a consumptive man, he had been unable, by injection into the peritoneal cavity of bovines, to reproduce the disease; and while tuberculisoidin and other antitoxins have marked beneficial effect upon cases of the disease which resulted from the inoculation of the bacilli from man, they had no such salutary effect when injected into animals which had derived the disease from the bovine virus. This was confirmed by experiments on three guinea-pigs which had been inoculated with bovine virus and were afterward treated with one of these preparations furnished by Dr. E. Klebs. After the disease was well advanced the treatment had not the slightest effect upon its progress. The same results were obtained by Czaplewski and Roloff in 1892 (*Berlin Klin. Wochenschrift*, No. 29). While these experiments may not be conclusive, they all point in the same direction.

The Commission has had a number of remedies offered which were either secret (and on trial found to be useless) or were made of drugs whose action is already well known. The latest of these are in the nature of the so-called antitoxins, to the action of which on the bovine virus I have already called your attention. Curative influence with these agents has been noted only in cases inoculated with the weakened laboratory cultures, and, even if they were distinctly servicable in the early stages of the disease, they would be entirely valueless to us because of the impossibility of detecting the extent of tuberculosis in any given case by the means at our command.

Tuberculosis differs from other infectious diseases principally in the degree of its activity, being generally of long duration. The infection may be distributed, as is well known, by most of the secretions and excretions of the animal economy, hence the danger that exists in the immediate surroundings of the infected animal. It is a recognized fact that in the animal body alone do the bacilli multiply; therefore, if these animals are removed and the stables thoroughly disinfected, we are safe in concluding that the ravages of the disease will be very materially checked. It can only be detected in the early stage and in many advanced stages by the aid of tuberculin; therefore, this agent has become indispensable in diagnosis. It not only reveals the disease in its incipiency, but in its gravest and most dangerous types; it does not, however, as a rule, discriminate between these cases.

This leads us to the question, What shall be done with the affected animals? Our statutes provide that all known cases of the disease shall be destroyed and the carcasses disposed of otherwise than for food. It has been urged by the agricultural economist that animals only slightly affected and not showing the disease on physical examination might be separated from the herd, and their product, either of meat or milk, utilized. This might be were it not for the danger to consumers; but here the interests of the public are diametrically opposed to those of the agriculturist, for it is now an accepted fact that milk from tuberculous udders generally contains the bacilli in greater or less number, and that they are frequently present in the milk from udders which show no appreciable lesion on careful post-mortem examination. The question of the danger from this product is apparent when we consider that it is largely used by those who are especially susceptible. Again, if we could distinguish those cases where a single gland is affected, this danger from the milk-supply could be easily removed; but our experience has demonstrated that in many instances it is impossible upon physical examination to determine whether an animal is tuberculous, whether it is extensively tuberculous, or whether it is entirely free from the disease; hence the suspicion which rests on milk from untested herds.

The aim of the Commission in Massachusetts has been to advise legislation, based upon their present knowledge of the disease, that should not be oppressive to the agricultural community, but at the same time safely guard the interests of the public health. The action of our Legislature resulted in the passage of a bill, which was signed by the Governor on June 5, 1895, removing from individual owners any hardships, in that it provided for the payment from the treasury of the Commonwealth for the value of all tuberculous animals condemned by this Commission; but it left it optional with the owners whether they would take advantage of tuberculin as a diagnostic or not. It thus failed to afford any protection to the tax-paying consumer, since it left the eradication of the disease from the herds furnishing our city milk-supply entirely at the option of the producer.

It has been but a short time since we were able to detect with any accuracy the presence of tuberculosis in the bovine species, but now having an agent that will reveal the disease in its earliest forms are we justified in advocating measures which leave the eradication of the disease to the option of interested owners? And, further, are we justified in continuing to allow the milk-product from animals to be placed upon the market without certification of the animal's health, based on tuberculin? Our legislative bodies have passed stringent regulations as to the adulteration of almost all food-supplies, extending the same to cover the introduction of foreign material into the milk as a preservative; but they hesitate to enact and inforce stringent laws that shall apply to the marketing of milk which is not free from suspicion of disease. It is not my wish, for a moment, to convey to you the idea that I believe the milk-supply is responsible for the greater part of tuberculosis in the human family; I simply desire to point out that it is an important element of danger that can and should be removed.

Sanitation has already exerted a great influence in the reduction of the mortality statistics in the human family, and similar laws should be enacted regarding the conditions under which animals are allowed to be kept. Owing to the prejudice that many farmers have against the admission of fresh air into their stables the atmosphere is often particularly foul, being permeated with the organic emanations from the animal. I believe that if the extent of the loss of life among animals in this country, due directly or indirectly to confinement in an impure atmosphere, were obtainable, the results would be sufficiently appalling to call for immediate legislation. Men should not, either from ignorance or indifference, be allowed to shut their animals up in places not big enough for a human being to live in, or to make them breathe contaminated air, either through their personal ignorance or carelessness. It is no argument that because it is his own property a man is at liberty to do with it as he chooses. Certainly not if he markets the products from such animals. Why should a man be allowed to run the risk of communicating disease to the human family or to other animals with which they come in contact? Our laws provide a penalty for selling a glandered horse or leading him over the public highway. Why should it not provide a penalty for owners who refuse to provide their animals with such sanitary surroundings as science dictates they require? No person should be allowed to breed disease among his own animals or risk the property of others and the lives of human beings.

In the city of Boston there is scarcely a week passes that prosecutions are not instituted and convictions secured for the sale of milk that is deficient in solids (in the majority of cases to be accounted for by the addition of water), while at the present time there is no law in the Commonwealth to prevent the owner of an animal affected with tuberculosis (providing such diagnosis could not be made upon physical examination) from marketing the milk from such animal, and no restrictions whatever to the importation of milk from beyond State lines. Many of our Western cities have already instituted regulations providing that parties desiring to sell milk within their limits shall not receive a license so to do until they have proved by a certificate of tuberculin-test that their animals are free from tuberculosis; and the city of Rome has a similar restriction. With the wild West and the most typical of the effete nations of Europe thus joining hands in the protection of public health, is it not time for our enlightened Eastern cities to agitate the question continually until at least equally efficient measures are adopted here? If the State cannot institute in its legislative halls regulations providing for a pure milk-supply it certainly devolves upon the health officers of municipalities to make regulations sufficiently stringent to protect the consumer from such a suspicious product as that obtained from milch-cows throughout our New England States until such time as they have been pronounced free from disease by the tuberculin-test.

*Under Law of* 1894, *to December* 15, 1894. Inspectors quarantined 3295; condemned 810, or 24.58 per cent. At quarantine stations, 1432; condemned 89, or 6.21 per cent. Nantucket (systematic), 665; condemned 6, or 0.9 per cent. Total, 5392 tested and 905 condemned.

*From December* 15, 1894, *to January* 5, 1895. Inspectors quarantined 1776; condemned 795, or 44.8 per cent. Systematic inspection of 3500; condemned 6, or 0.17 per cent. Quarantine stations, 6270; condemned 251, or 4 per cent. Interstate cattle, 314; condemned 5, or 1.6 per cent. Veterinarians (private tests), 1518; condemned 289, or 19 per cent. Total, 13,378 tested and 1346 condemned.

*Laws of* 1895, *June* 5 *to November* 5, 1895. Local inspectors quarantined 1243; condemned 643, or 52 per cent. By voluntary request, 1894; condemned 510, or 27 per cent. Veterinarians (private tests), 504; condemned 90, or 17.3 per cent. Quarantine stations, 900; condemned 35, or 3.9 per cent. Interstate cattle, 400; condemned 11, or 2.8 per cent. Errors due to tuberculin-test since June 5th, 0.1 per cent.

*Compensation.* Under the law of 1894, 2250 animals were destroyed; average, $21 per head (half appraisal); total, $47,000. Under the law of 1895, 1200 animals were destroyed; average, $36 per head (full appraisal); total, $41,000. Returned directly to the farmers, $88,000.

